# Supplementary material for: Functional Properties of Corn Byproduct-Based Emulsifier Prepared by Hydrothermal–Alkaline
Source: Molecules. 2023 Jan 9;28(2):665. doi: 10.3390/molecules28020665 (PMC9865437; doi:10.3390/molecules28020665)
Supplement: Supplementary file 1 [file molecules-28-00665-s001.zip › molecules-2062131-supplementary.pdf]

Evaluation of byproduct-based emulsifier by hydrothermal-alkaline

Supplementary materials

Table S1. Recipe for two pieces of 1.5-mm thick plywood

	16.50% Separating gel	10% spacer gel	4% stacking gel
AB-3 stock solution	/	1.221 mL	0.48 mL
AB-6 stock solution	4.5 mL	/	/
Gel buffer	4.5 mL	0.667 mL	0.496 mL
50% glycerol	2.88 mL	/	/
ddH_2O	1.62 mL	2.778 mL	4.032 mL
10% AP	120 μL	60 μL	60 μL
TEMED	15 μL	9 μL	9 μL

AB: acrylamide-bisacrylamide; AP: ammonium persulfate; TEMED: tetramethyl ethylenediamine

Table S2. Average particle size of emulsions stabilized by $ZH_{0.6\text{-}70}$ after adding different concentrations of NaCl (n=3)

Samples	$D_{[3,2]}$/μm	SSA/m^2/kg
$ZH_{0.6\text{-}70}$—0mM	1.26 ± 0.02^{c}	4775.8 ± 11.88^{b}
$ZH_{0.6\text{-}70}$—10mM	1.26 ± 0.03^{c}	4756.4 ± 20.85^{b}
$ZH_{0.6\text{-}70}$—50mM	1.24 ± 0.01^{c}	4932.2 ± 8.35^{a}
$ZH_{0.6\text{-}70}$—100mM	1.40 ± 0.04^{b}	4296.6 ± 15.08^{c}
$ZH_{0.6\text{-}70}$—200mM	1.47 ± 0.03^{b}	4093.4 ± 21.08^{d}
$ZH_{0.6\text{-}70}$—500mM	2.34 ± 0.02^{a}	2561.2 ± 22.91^{e}

SSA: specific surface area.

* Different superscript letters in same column indicate significant difference ($P < 0.05$).

Table S3. Average particle size of emulsions stabilized by $ZH_{0.6-70}$ at different temperature (n=3)

Samples	$D_{[3,2]}$/μm	SSA/m^2/kg
$ZH_{0.6-70}$—0h	1.50 ± 0.01[a]	4893.40 ± 12.42[a]
$ZH_{0.6-70}$—70℃	1.51 ± 0.02[a]	4792.33 ± 166.71[ab]
$ZH_{0.6-70}$—80℃	1.47 ± 0.03[a]	4744.80 ± 22.40[b]
$ZH_{0.6-70}$—90℃	1.48 ± 0.02[a]	4026.20 ± 5.40[c]

SSA: specific surface area.

* Different superscript letters in same column indicate significant difference (P < 0.05).

Table S4. Average particle size of emulsions stabilized by $ZH_{0.6-70}$ at different pH (n=3)

Samples	$D_{[3,2]}$/μm	SSA/m^2/kg
$ZH_{0.6-70}$—initial	1.23 ± 0.03[e]	4893.40 ± 12.42[a]
$ZH_{0.6-70}$—PH=9	1.33 ± 0.05[de]	4792.33 ± 166.71[fg]
$ZH_{0.6-70}$—PH=8	1.26 ± 0.02[d]	4744.80 ± 22.40[f]
$ZH_{0.6-70}$—PH=7	1.49 ± 0.01[d]	4026.20 ± 5.40[e]
$ZH_{0.6-70}$—PH=6	2.48 ± 0.05[c]	2421.60 ± 50.24[d]
$ZH_{0.6-70}$—PH=5	9.88 ± 1.13[b]	613.34 ± 68.99[c]
$ZH_{0.6-70}$—PH=4	138.2 ± 15.22[a]	43.88 ± 4.70[a]
$ZH_{0.6-70}$—PH=3	147.6 ± 17.81[a]	41.19 ± 4.82[a]

SSA: specific surface area.

* Different superscript letters in same column indicate significant difference (P < 0.05).

Table S5. Volumetric mean diameters of four kinds of emulsions during storage (n=3)

Samples	0d (μm)	15d (μm)	29d (μm)
$ZH_{0.6-70}$	1.33±0.02[b]	1.41±0.01[b]	13.34±3.73[a]
NaCN	2.28±0.01[b]	2.27±0.01[b]	18.43±4.11[a]
E322	2.54±0.01[c]	6.60±0.16[b]	50.64±17.92[a]
E473	5.66±0.02[b]	5.34±0.02[b]	18.54±4.80[a]

NaCN: sodium caseinate; E322: soybean lecithin; E473: sucrose esters of fatty acids.

* Different superscript letters in same row indicate significant difference (P < 0.05).

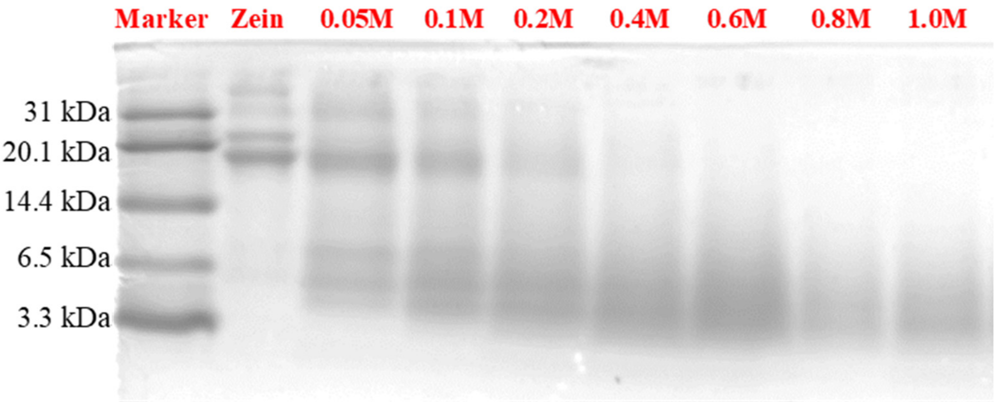

Figure S1. Electrophoretogram of zein hydrolysates (ZHs) obtained from treatment with different concentrations of alkali

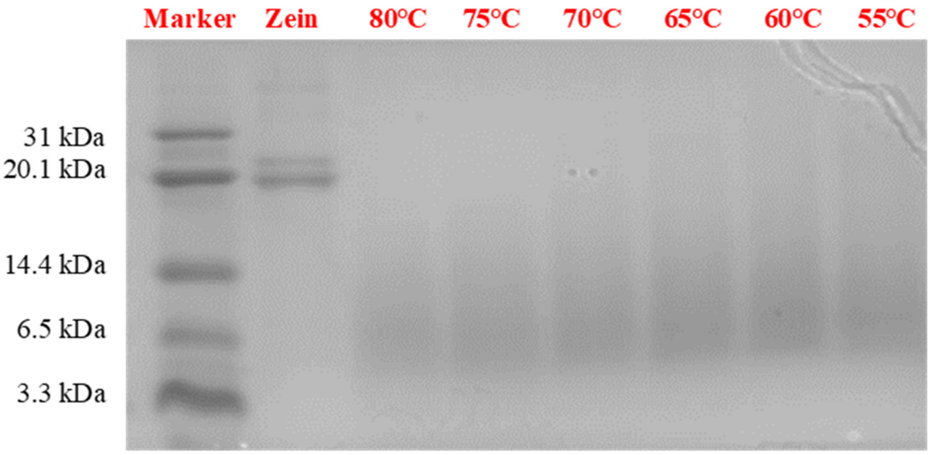

Figure S2. Electrophoretogram of ZHs obtained from treatment with different temperatures

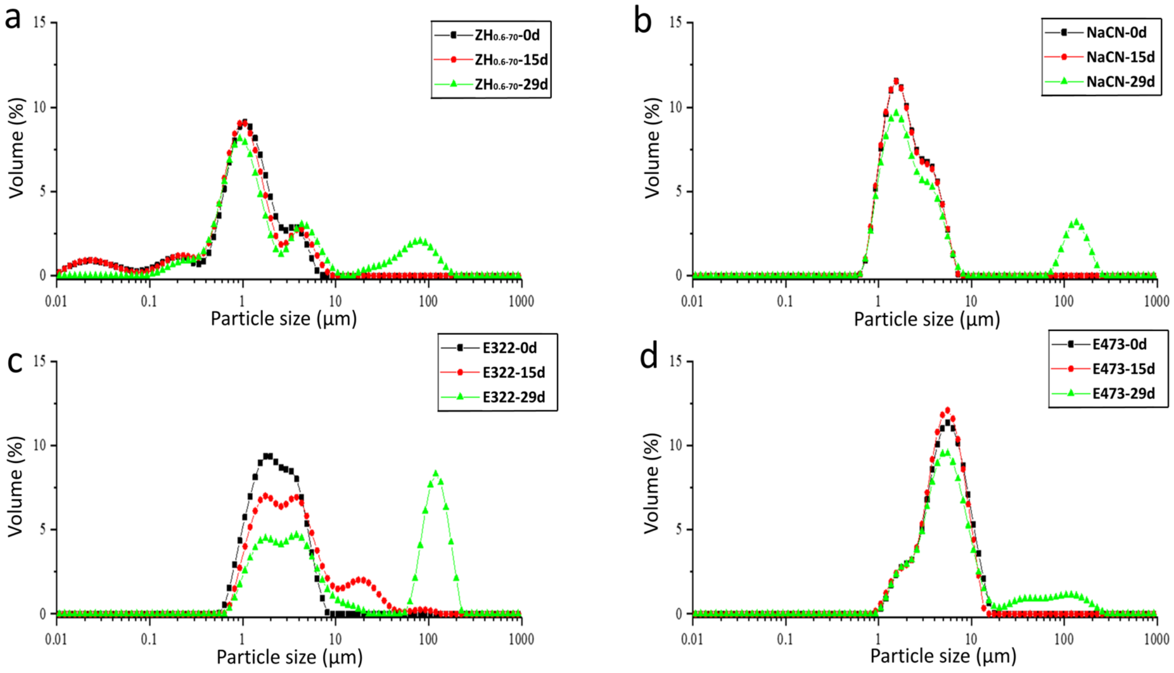

Figure S3. Particle size distribution of emulsions stabilized by $ZH_{0.6\text{-}70}$ (a), sodium caseinate (NaCN, b), soybean lecithin (E322, c) and sucrose esters of fatty acid (E473, d) during 29 days of storage at 25 °C

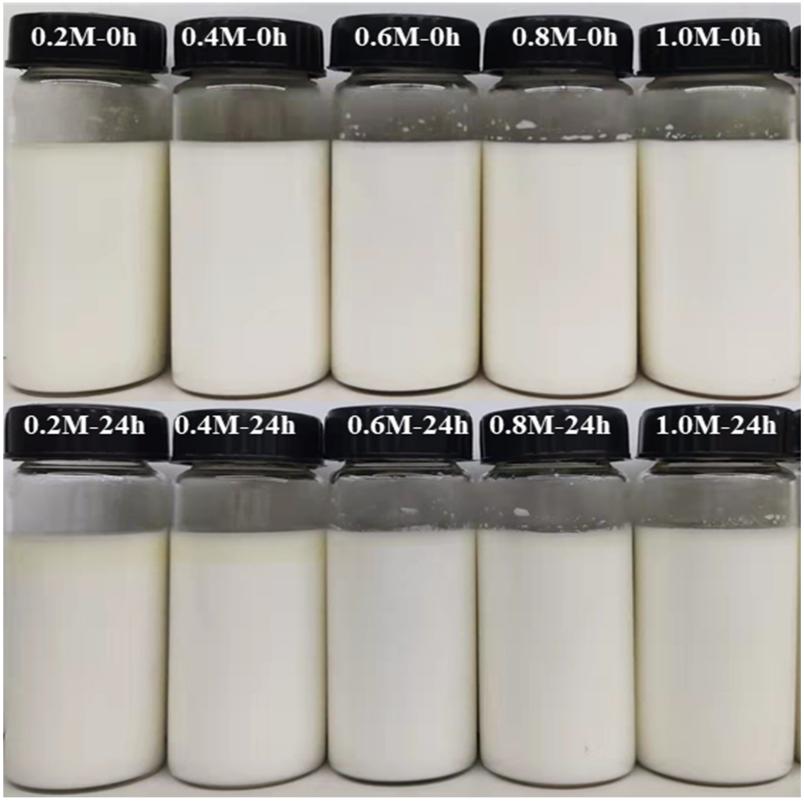

Figure S4. Pictures of emulsions made by zein hydrolysates obtained from treatment with different concentrations of alkali

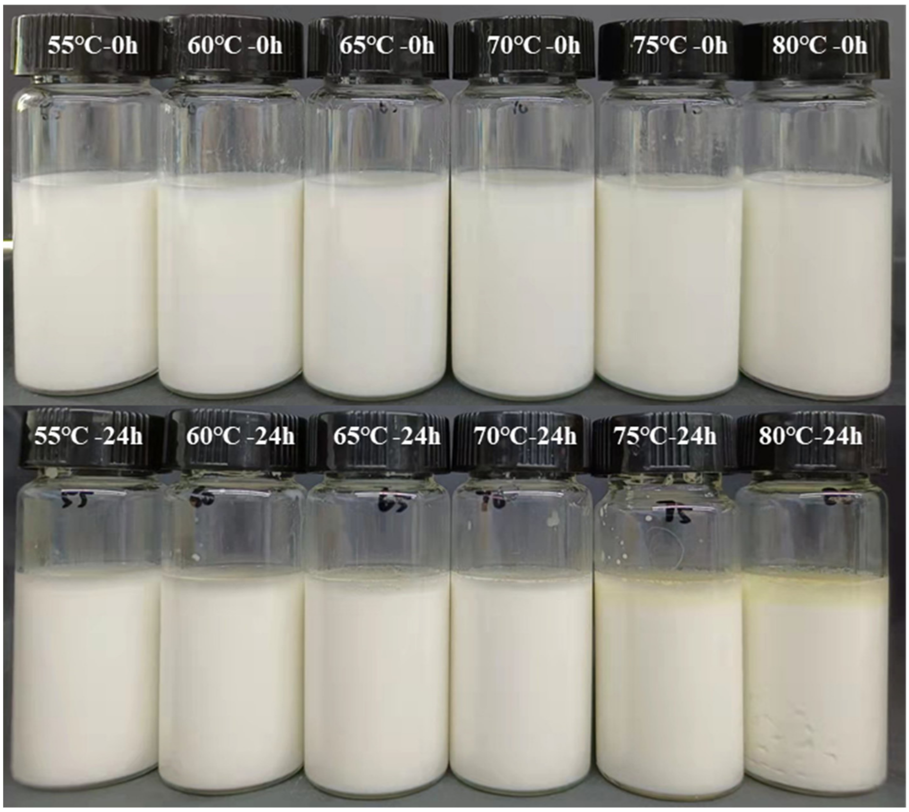

Figure S5. Pictures of emulsions made by zein hydrolysates obtained from treatment with different temperatures
